# Radiometric Calibration of an Inexpensive LED-Based Lidar Sensor

**DOI:** 10.3390/s20185215

**Published:** 2020-09-13

**Authors:** Jordan Laughlin, Preston Hartzell, Craig Glennie, Jan W. Kovermann

**Affiliations:** 1 United States Military Academy, West Point, NY 10996, USA; jordan.laughlin@westpoint.edu; 2 Department of Civil and Environmental Engineering, University of Houston, Houston, TX 77204, USA; clglennie@uh.edu; 3 Terabee, 01630 Saint-Genis-Pouilly, France; jan.kovermann@terabee.com

**Keywords:** lidar, radiometric calibration, neural networks

## Abstract

Radiometric calibration of laser-based, topographic lidar sensors that measure range via time of flight or phase difference is well established. However, inexpensive, short-range lidar sensors that utilize non-traditional ranging techniques, such as indirect time of flight, may report radiometric quantities that are not appropriate for existing calibration methods. One such lidar sensor is the TeraRanger Evo 60 m by Terabee, whose reported amplitude measurements do not vary smoothly with the amount of return signal power. We investigate the performance of a new radiometric calibration model, one based on a neural network, applied to the Evo 60 m. The proposed model is found to perform similarly to those applied to traditional lidar sensors, with root mean square errors in predicted target reflectance of no more than ±6% for non-specular surfaces. The radiometric calibration model provides a generic approach that may be applicable to other low-cost lidar sensors that report return signal amplitudes that are not smoothly proportional to target range and reflectance.

## 1. Introduction

Radiometric calibration of lidar intensity to physical units such as relative reflectance is commonly practiced in the remote sensing community to extract target spectral information at the wavelength of the lidar light source [1,2,3,4,5,6,7]. The lidar sensors employed by the remote sensing community traditionally use a laser light source and a pulsed time of flight or phase difference range measurement method [8,9]. These sensors are typically expensive to purchase and maintain, making recent developments in economical lidar sensors based on alternative range measurement methods (e.g., structured light, indirect time of flight (ITOF)) a potentially attractive option for topographic remote sensing. However, little work has investigated the radiometric calibration of these sensors, perhaps due to their relatively short ranges (often less than a few tens of meters, e.g., [10,11]) and performance challenges in high ambient light environments.

Recently, an LED-based lidar sensor with an advertised range of up to 60 m and reported centimeter-level precision was made commercially available—the TeraRanger Evo 60 m by Terabee [12]. The sensor is economically priced under $150 and, among other observables, reports a return signal amplitude measurement. These characteristics make the Evo 60 m a candidate for affordable, simultaneous acquisition of target range and reflectance if a suitable radiometric calibration model can be developed. However, a key difference between the Evo 60 m and traditional laser-based lidar sensors is the use of an ITOF ranging method. Although the ITOF method does not preclude radiometric calibration of the return signal amplitude, e.g., see [13], the ITOF method employed by the Evo 60 m features an automatically varying integration time, which is the length of time the optical power returned from the Evo 60 m’s LED light source is collected to generate a range measurement. If the return signal power is low, the integration time increases to generate a more reliable range measurement, i.e., to improve the signal-to-noise ratio. Ambient light levels also influence the integration time, which changes in discrete steps rather than a continuous manner. The variable integration time directly influences the reported return signal amplitude, resulting in large amplitude discontinuities that correspond to changes in ambient light, target reflectance, and target range. The complex relationships between the reported amplitudes, target and environmental properties, and sensor response characteristics challenge the ability to apply traditional physical and empirical lidar radiometric calibration models to the Evo 60 m measurements and, therefore, motivate the development of a new approach.

We investigate a radiometric calibration model based on a simple neural network that empirically approximates the relationship between target reflectance and the measurements reported by the Evo 60 m: return signal amplitude, target range, ambient light level, and measurement integration time. The proposed method is generic, and may therefore be extensible to other low-cost lidar sensors that do not report traditional, smoothly varying amplitude (a.k.a. intensity) values. In addition to investigating the performance of the proposed radiometric calibration model, we also examine the consistency of the Evo 60 m reflectance estimates over time and between sensors to better evaluate the potential of the sensor to produce dependable radiometric information.

The remainder of this paper is organized as follows. We review traditional lidar radiometric calibration approaches to further motivate this study and the ITOF ranging method in Section 2. Descriptions of the TeraRanger Evo 60 m lidar sensor, the calibration and test targets used in this work, the proposed radiometric calibration model, and the methods used to collect measurements and train the proposed model are given in Section 3. The performance of the proposed calibration model is discussed in Section 4 and conclusions summarized in Section 5.

## 2. Background

### 2.1. Lidar Radiometric Calibration

For a traditional, laser-based lidar sensor, the power received, $P_{r}$, by the sensor is related to the physical characteristics of the illuminated target, acquisition geometry, the atmospheric environment, and system characteristics. This relationship, often termed the lidar range equation, is given in [14] as:

(1) $$P_{r}=\frac{P_{t}D_{r}^{2}}{4\pi R^{4}\beta_{t}^{2}}\eta_{sys}\eta_{atm}\sigma ,$$

where $P_{t}$ is the transmitted power, $D_{r}$ is the diameter of the receiver aperture, *R* is the target range, $\beta_{t}$ is the laser beam width, $\eta_{sys}$ is the system transmission factor, $\eta_{atm}$ is the atmospheric transmission factor, and σ is the target cross section given as:

(2) $$\sigma =\frac{4\pi }{\Omega }\rho A_{t},$$

where Ω is the solid angle of scattering, ρ is target reflectance, and $A_{t}$ is the target area.

Under the assumptions of a Lambertian target that intercepts the entirety of the transmitted power, a negligible atmospheric transmission factor due to short ranges, constant system parameters, and normal target incidence, Equations (1) and (2) can be combined and simplified to:

(3) $$P_{r}=\frac{\rho }{R^{2}}C_{cal},$$

where $C_{cal}$ is a calibration parameter containing constant terms from Equations (1) and (2). See [14] for a step-by-step derivation of how the assumptions noted above lead to this simplified form of the lidar range equation. Solving for target reflectance and inverting the value within $C_{cal}$ leads to:

(4) $$\rho =C_{cal}P_{r}R^{2}.$$

Thus, under reasonable simplification, target reflectance is proportional to received optical power and target range. If one or more targets of known reflectance are observed, the calibration constant, $C_{cal}$, can be computed and target reflectance can be estimated for subsequent lidar measurements where both target range and return signal power measurements are reported.

Equations (1)–(4) assume that the return signal power measurement is linear with respect to the actual power incident on the sensor. This characteristic is common among airborne lidar sensors, and the above equations can be used to radiometrically calibrate these sensors [4,14]. However, in contrast to airborne lidar, terrestrial lidar sensors must observe both very close and distant targets. Their response to such a broad range of returned optical power can be non-linear, and a single calibration constant ($C_{cal}$) may not be sufficient. However, their system parameters are still constant, and therefore their reported return signal intensities vary smoothly, although non-linearly, with the amount of received optical power. Thus, empirical functions or interpolation from look up tables (LUTs) can be used to model the relationship between target reflectance and the reported target range and intensity [5,15].

For the ITOF-based Evo 60 m lidar sensor examined in this paper, the system parameters are not constant—the integration time is variable. This negates the ability to use a physical model with a single calibration constant, or the use of a single empirical functional approximation or LUT. We observed 12 different integration times in our work with the Evo 60 m, which would require 12 values for $C_{cal}$ assuming the sensor has a linear response, or 12 functional approximations or LUTs for a nonlinear sensor. Experimentation with generating unique $C_{cal}$ values for each integration time did not produce stable results, suggesting a non-linear sensor response. Comparisons of range, amplitude, and ambient light values of measurements having a common integration time also yielded complex relationships not easily modeled with functions or LUTs. These observations motivate our pursuit of an empirical approximation in the form of a simple neural network that is capable of estimating target reflectance values from measurement data that spans all integration times.

### 2.2. Indirect Time of Flight

Although not required for understanding the proposed radiometric calibration method, we briefly review the ITOF ranging technique. Radiometric calibration of lidar intensity is primarily of interest to the remote sensing community, which is most familiar with pulsed time of flight and traditional phase difference lidar techniques, and a review will provide context to the work herein.

**Figure 1:**
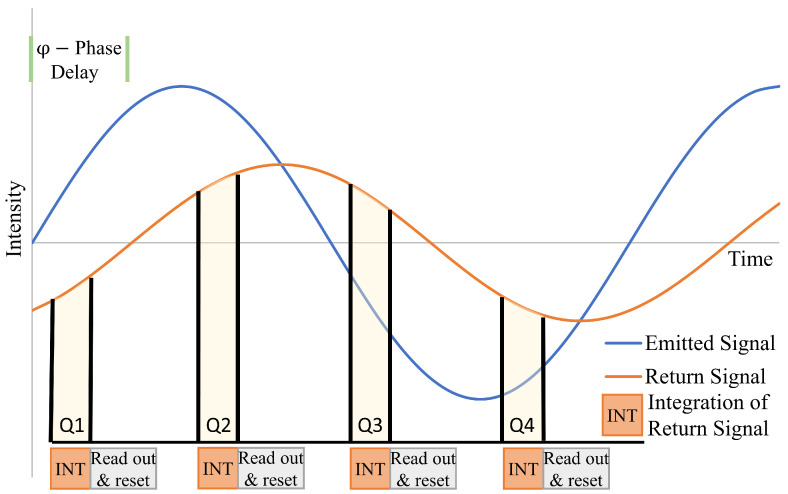
Quadrature sampling illustration. The return signal amplitude is attenuated due to target range and reflectance. For clarity, the return signal is shown centered on the same horizontal axis as the emitted signal (background light is ignored).

An ITOF lidar sensor continuously emits amplitude modulated light, similar to traditional laser-based phase difference lidar sensors. However, instead of measuring phase delay through direct comparison or mixing of the return signal with a version of the emitted signal, ITOF relies on quadrature sampling of the return energy [16,17]. The amount of return energy is integrated four times per period, i.e., at 90-degree phase intervals (see Figure 1), the timing of which is aligned with the phase of the emitted signal. The measure of phase of the return signal, φ, is thus the phase delay between the emitted and return signal and is computed from the integrated quadrature energy samples as:

(5) $$\varphi =\tan^{-1}\frac{\left(Q_{3}-Q_{1}\right)}{\left(Q_{4}-Q_{2}\right)},$$

where $Q_{1}$ through $Q_{4}$ are the quadrature samples of integrated return energy. Target range is then calculated using the standard equation for phase difference lidar:

(6) $$z=\frac{1}{2}\cdot \frac{c}{f}\cdot \frac{\varphi }{2\pi },$$

where *c* is the speed of light and *f* is the frequency of the modulating wave. Note that, as with traditional continuous wave phase difference lidar, the maximum unambiguous target range is half the length of the modulating wavelength.

The amplitude, or intensity, *A*, of the return signal can also be computed from the integrated signal measurements as:

(7) $$A=\frac{\sqrt{{\left(Q4-Q2\right)}^{2}+{\left(Q1-Q3\right)}^{2})}}{2}.$$

Finally, the background light, *B*, which is the sum of ambient light and the DC component of the attenuated light reflected back from the target, is calculated as:

(8) $$B=\frac{\left(Q1+Q2+Q3+Q4\right)}{4}.$$

For the Evo 60 m, the integration time automatically increases when observing low reflectance targets, i.e., when the return energy is low. Although the exact details are unknown, a reasonable conjecture—based on the observation that the rate at which the measurements are reported decreases with increasing integration times—is that that multiple quadrature measurements are summed to improve the signal to noise ratio. Thus, as has been observed, reported amplitude measurements can sometimes increase when observing successively lower reflectance targets at the same range.

## 3. Materials and Methods

### 3.1. Materials

#### 3.1.1. Sensor

The Terabee TeraRanger Evo 60 m is a LED-based lidar sensor that uses an ITOF ranging method. The Evo 60 m provides distance measurements in its standard configuration. By using a debugging command along with custom firmware provided by Terabee, the sensor reports additional measurements of return signal amplitude, ambient light, and integration time for a total of four observables. The basic specifications for the Evo 60 m are given in Table 1. Note that the maximum range of 60 m is only achieved in optimal conditions, i.e., when observing a highly reflective target in low ambient light conditions [18].

The specifications indicate a change in the resolution (quantization) of the reported range and a change in accuracy at 14 m. Testing with several Evo 60 m sensors indicates that this threshold indeed exists and imparts both a distinct change in range precision and, for the sensors we tested, a range bias of ∼10 cm. Given the presence of these changes at 14 m, along with a maximum range of 18 m within the laboratory used for calibration data collection, the maximum range of the radiometric calibration model developed in this work was limited to 14 m.

Four Evo 60 m sensors were used throughout the work to test the consistency of the measurements and radiometric calibration results between multiple sensors. A single Evo 60 m was purchased in May 2018 and three additional sensors were purchased in May 2019.

#### 3.1.2. Calibration and Test Targets

Two groups of homogeneous, flat materials were used as targets for generating and subsequently testing the radiometric calibration models developed for the Evo 60 m sensors. Due to the large sensor field of view (FOV) of 1.7 degrees, target size is an important consideration. Table 2 shows the field of view diameter for the Evo 60 m from 1 to 60 m in range.

**Figure 2:**
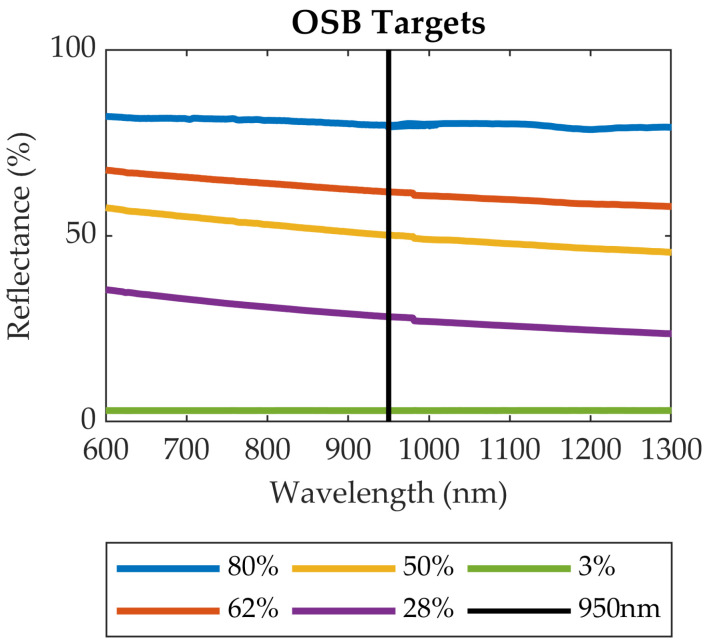
Spectral signatures of the OSB calibration targets. The vertical black line indicates the wavelength (950 nm) of the Evo 60 m LED. The target reflectance values at 950 nm are shown in the legend.

The first group of targets was used to generate the radiometric calibration models (training the neural networks). This group consisted of five reflectance standards constructed from 4 × 4 foot (1.2 × 1.2 m) square panels of oriented strand board (OSB) painted with multiple mixtures of white and black matte (flat) latex paint. The large panel dimensions comfortably accommodate the sensor FOV up to 20 m range, and the matte paint finish was selected to approximate Lambertian scattering characteristics. The spectral signatures of the painted OSB targets were measured with a Spectra Vista Corporation (SVC) spectroradiometer (model HR-1024 with 1024 channels and a range of 350–2500 nm) using a Spectralon^®^ 99% reflectance standard as the white reference. Figure 2 shows the spectral signature of the OSB calibration targets, which are hereafter differentiated by their reflectance values at the 950 nm LED wavelength of the Evo 60 m sensor: 3%, 28%, 50%, 62%, and 80%.

The performance of the radiometric calibration models was tested on a second group of targets consisting of 12 flat surface, ‘real-world’ materials. Seven of the targets were indoor surfaces; the remaining five targets were outdoors. As with the OSB calibration panels, the reflectance of each test target at 950 nm was measured using an SVC spectroradiometer (Table 3).

### 3.2. Methods

#### 3.2.1. Sensor Alignment

Unlike a laser scanner, the Evo 60 m does not provide a point cloud that can be examined for spatial structure, nor does it provide a visual indicator such as a reference dot or an aligned sight. Given that many of the targets to be illuminated by the Evo 60 m are not large (i.e., not all targets will be a wall several meters in width and height), knowledge of where the sensor field of view is aimed is important. A custom mount was therefore constructed that enables the alignment of the four Evo 60 m sensors with a Leica Disto laser distance meter (model E7400x), which emits a visible red laser beam.

**Figure 3:**
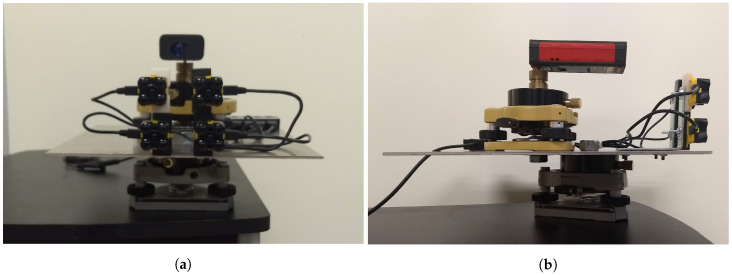
(**a**) Front view and (**b**) side view of the alignment device constructed from an aluminum plate, two survey tribrachs, a Leica Disto (acting as the range measurement standard and sighting laser), and various connection hardware.

The mount was constructed from an aluminum plate, two survey tribrachs, two angle brackets, four 3D-printed sensor brackets, and various attachment hardware as shown in Figure 3. The mount not only maintains the relative alignment of the four sensors to the laser distance meter, but also provides the ability to adjust the horizontal and vertical viewing angles of the entire sensor suite via the bottom tribrach. The mount is attached to a small rolling desk, allowing it to be easily moved to multiple ranges from a static target. After assembling the mount, the sensors were aligned with the Leica Disto at a range of seven meters using an infrared-sensitive camera in a limited light environment. Seven meters was the maximum range at which the LED light pattern emitted by the Evo 60 m sensors could be observed with the infrared camera.

#### 3.2.2. Data Collection

The targets were observed at different distances, ambient light levels, and incidence angles. Fifty measurements were collected for each unique combination of these variables, with each group of 50 measurements hereafter referred to as a ‘setup’. For each setup, the Leica Disto was used to align the sensor FOVs onto the target and also to record a reference range. The 50 measurements for each of the four Evo 60 m sensors were then collected in quick succession. All data collection occurred in a dry environment.

Four separate groups of data were collected. The first group was for training the four neural networks that serve as the radiometric calibration model for the four sensors and was therefore distributed in target reflectance, range, and ambient light levels. Measurements to the five OSB calibration panels were collected indoors at each meter of range between 1 and 18 m and at three ambient light levels using a halogen light source. Additionally, the OSB targets were observed outdoors at the same ranges but at very high ambient light levels (clear, sunny day). This data was collected in June 2019 and consists of 15,400 unique measurements (308 setups) per sensor.

A second group of data was collected for testing the trained radiometric calibration models. Similar to the procedure used for collecting the calibration data group, the seven indoor test targets were observed at each meter between 1 and 18 m with ambient light levels varied via a halogen light source. The five outdoor test targets were collected at the same ranges in ambient light levels that varied naturally according to solar conditions. This data was collected in June 2019 and consists of 15,000 unique measurements (300 setups) per sensor.

A third group of data was collected to examine the temporal stability of the sensors. This group is essentially a repeat of the second (test) group, but acquired two months later. Measurements to two of the OSB calibration targets, the 28% and 50% targets, were also acquired. This group was collected in August 2019 and consists of 25,600 unique measurements (512 setups) per sensor.

The last group of data was collected to qualitatively evaluate the Lambertian scattering characteristic of the OSB calibration targets and several test targets that produced anomalous results. Measurements were made with the incidence angle varied in ten-degree increments from 0 to 60 degrees. The collection was performed indoors. Since the effect of incidence angle was the focus, target distance and ambient light were held constant. The ambient light was kept at a low level to create a favorable environment for the sensor. This group of incidence angle data was collected in July 2019.

#### 3.2.3. Radiometric Calibration Model

As stated earlier, a radiometric calibration model based on an artificial neural network is investigated in this work to accommodate the complex relationships between the range, amplitude, ambient light, and integration time measurements reported by the Evo 60 m. A simple feedforward network with these four reported measurements as inputs and a single output (target reflectance) is used. Note that the range input is the range reported by the sensor, not the reference range measurement from the Leica Disto laser distance meter. The number of hidden layers, number of nodes within each layer, activation function, and network weight optimization algorithm were empirically chosen based on best performance. MATLAB^®^ was used for the network design and analysis. The number of hidden layers, activation function, and optimization algorithm were consistent for all four sensors. However, the number of nodes within each hidden layer was allowed to vary; thus, each sensor has a specific network architecture.

In order to train the the neural networks, the first group of observations (the OSB calibration panels) was split into train (90% of the data) and validate (10% of the data) sub-groups. Since the 50 measurements comprising each setup are not independent, they were not split between the train and validation groups, i.e., all 50 measurements within a setup were placed either in the train or validate sub-groups. The number of layers, number of nodes within each layer, activation function, and optimization algorithm were empirically chosen based on the performance of the trained networks on test data drawn from the second group of observations (‘real world’ targets). Ten percent of the setups in the second group were used for this purpose after excluding surfaces found to have specular properties. The remaining 90% of the second group was used for evaluating the performance of the final trained models, which is reviewed in Section 4.

The neural network design was guided by comparison of the mean error between the reflectance values predicted by the trained networks for the test targets and the reflectance values measured by the spectroradiometer. To begin, the top 10% performing networks from a collection of networks—where the number of layers varied between one and three, the optimization algorithm was either Bayesian Regularization (BR), Levenberg-Marquardt (LM), or Scaled Conjugate Gradient (SCG), the number of nodes within each layer was randomly set between 1 and 20, and a random activation function was used—were compared. The BR algorithm produced the best results and two hidden layers performed better than either one or three hidden layers. Using the BR algorithm and two hidden layers, the top 10% performing networks were then selected from a collection of networks that used a linear (positive linear, saturating linear, or symmetric saturating linear) or sigmoid (Elliot symmetric sigmoid, log sigmoid, or hyperbolic tangent sigmoid) activation function and a layer node number that varied between one and twenty. The tangent sigmoid performed best and was therefore chosen. The final step was to select the number of nodes within each of the two hidden layers. A grid search was used where the number of nodes in the first and second layers ranged from one to fifteen. The unique combination that produced the lowest error was chosen for each sensor (Table 4).

Note that although Sensor 2 shows a markedly different node count than the other three sensors, many node count combinations produced very similar results. Therefore, we also examine the use of a common network architecture for all four sensors, comprised of eight and four nodes in Layers 1 and 2, respectively.

## 4. Results and Discussion

### 4.1. Radiometric Calibration Performance

A performance summary for each sensor’s trained radiometric calibration model (neural network) is provided in Table 5, where calibration model predicted reflectance values are compared to those measured with a spectroradiometer. Accuracy is quantified by the mean difference (Δ) and the root mean square error (RMSE) between the measured spectroradiometer reflectance values and the reflectance values predicted by the calibration model. Specifically, the RMSE is computed as:

(9) $$RMSE=\sqrt{\frac{\sum_{i=1}^{N}{\left(\rho_{p_{i}}-\rho_{m}\right)}^{2}}{N}},$$

where $\rho_{p_{i}}$ is the *i*-th predicted target reflectance value, $\rho_{m}$ is the reflectance value of the target measured with the spectroradiometer, and *N* is the number of predicted target reflectance values being compared to the measured target reflectance value. Precision is quantified with the standard deviation of the predicted reflectance values (σ). Although we make no assumption on the underlying distribution of predicted values (we simply use the standard deviation as a measure of dispersion), the predicted reflectance residuals were qualitatively observed to be normally distributed. The values in Table 5 were computed from target measurements spanning ranges from 1 to 14 m and all ambient light conditions.

**Figure 4:**
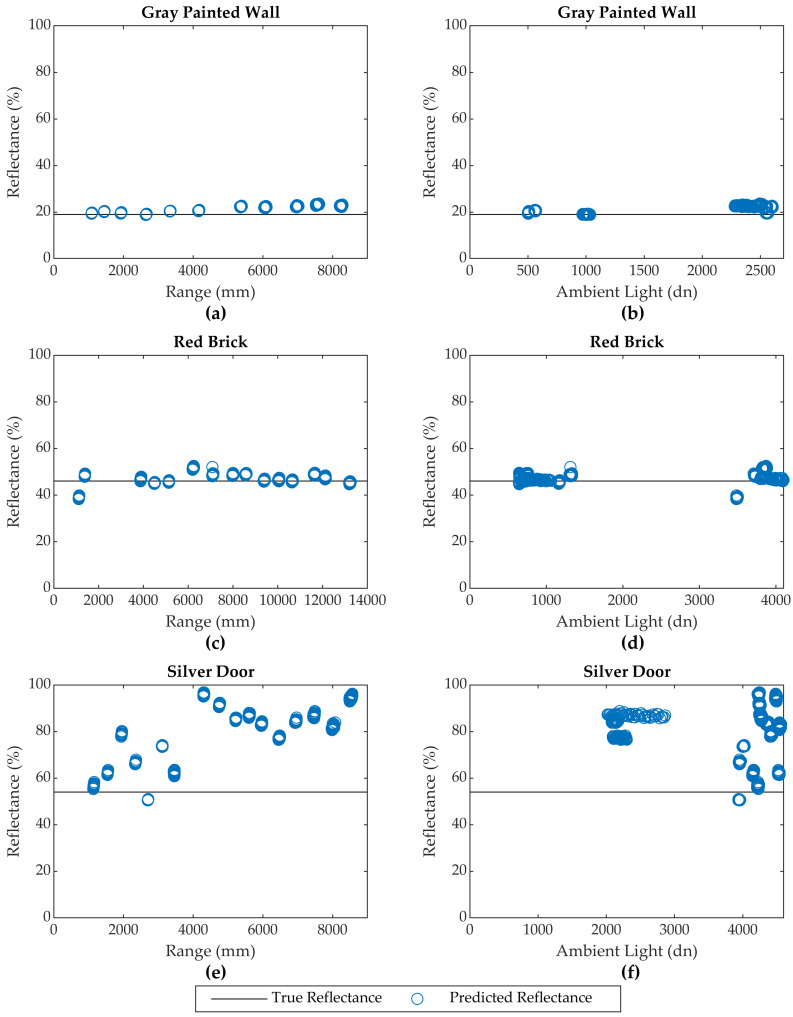
Consistency of predicted reflectance with respect to target range and ambient light levels for (**a**,**b**) the Gray Painted Wall, (**c**,**d**) Red Brick, and (**e**,**f**) Silver Door. Data from Sensor 1 shown.

With exception of three targets suspected of specular scattering (Silver Door, Brown Door, and White Painted Wall) and the Corkboard material, each calibrated sensor model predicted the test target reflectance values with mean differences of no more than 5% and RMSEs of no more than ±6%. The consistency of the predicted reflectance values throughout a span of different ranges and ambient light conditions is illustrated in Figure 4 for two typical targets: the Gray Painted Wall and Red Brick surfaces.

Except for the Corkboard target, those materials with poor reflectance predictions (Silver Door, Brown Door, and White Painted Wall) suffered from inconsistent results that varied with both range and ambient light. This is evidenced by their large standard deviations, which range from ±9% to over ±100% compared to a maximum of ±5% for all other targets. The inconsistencies in predicted reflectance versus target range and ambient light is illustrated for the Silver Door target in Figure 4. These materials also exhibit a high positive mean difference, which is believed to be caused by specular reflection. The Corkboard target, however, is anomalous. It exhibits a low standard deviation but suffers from a large negative mean difference. The scattering characteristics of several of the materials suspected of specular reflectance and the Corkboard are examined in the following section.

In terms of statistical significance, for almost all comparisons—43 out of 48—the difference between the mean predicted reflectance and the spectroradiometer measured reflectance is statistically significant at 2σ according to *t*-tests. This is not surprising, as reflectance estimates generated from lidar measurements are rarely in exact conformance with passive spectroradiometer measurements, e.g., see [3,5]. This is caused, in part, by the different viewing geometry of active lidar sensors from typical passive sensors. Lidar sensors observe targets at approximately the same geometry at which they are illuminated, while passive sensors typically view target surfaces at a different angle than the direction of illumination. This causes differences in phenomena such as shadow hiding that influence the amount of optical power returned back to the sensor [19].

#### 4.1.1. Incidence Angle Effect

**Figure 5:**
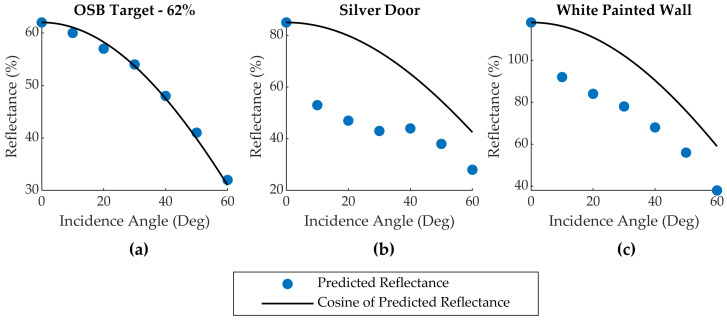
Reflectance values predicted by the radiometric calibration model (blue dots) and by Lambert’s Law (solid black lines) versus target incidence angle for the (**a**) 62% OSB Target, (**b**) Silver Door, and (**c**) White Painted Wall. The solid black line in each panel is the cosine of the incidence angle multiplied by the reflectance value predicted by the radiometric calibration model at zero degrees incidence. Data from Sensor 1 shown.

Three targets (Brown Door, Silver Door, and White Painted Wall) were suspected of specular reflectance based on their inconsistent and high predicted reflectance values compared to the spectroradiometer measurements (Table 5). This hypothesis was qualitatively investigated for the Silver Door and White Painted Wall targets by comparing the reflectance values predicted by the sensor radiometric calibration models to the reflectance value predicted by Lambert’s Law [20] for a series of measurements at different incidence angles. Lambert’s Law states that the amount of light reflected by a diffuse (non-specular) target is proportional to the cosine of the incidence angle. The 50% and 62% OSB reflectance targets and the Corkboard target were also examined. Graphs of the reflectance values predicted by Sensor 1’s calibration model for the 62% OSB target, Silver Door, and White Painted Wall versus those predicted by the cosine relationship in Lambert’s Law are given in Figure 5.

The 50% and 62% OSB calibration targets were found to follow the cosine trend predicted by Lambert’s Law for a diffusely scattering target, whereas the targets suspected of specular reflection exhibit a strong peak at 0-degree incidence, indicating the presence of specular scattering at near-normal observation geometries. The variation in the predicted reflectance for the specular targets (see Figure 4) are believed to result from small inconsistencies between setups in sensor alignment with respect to the targets, which produced small changes in incidence angle, as well as the large-scale roughness characteristics of some of the targets (e.g., the Silver Door target has a corrugated geometry). A similar incidence angle analysis was performed for the Corkboard target, which produced predicted reflectance values closely following a Lambertian cosine trend. Unfortunately, this does not provide any insight into why the radiometric calibration models predict much lower reflectance values for this target compared to the spectroradiometer measurement.

#### 4.1.2. Temporal Stability

**Figure 6:**
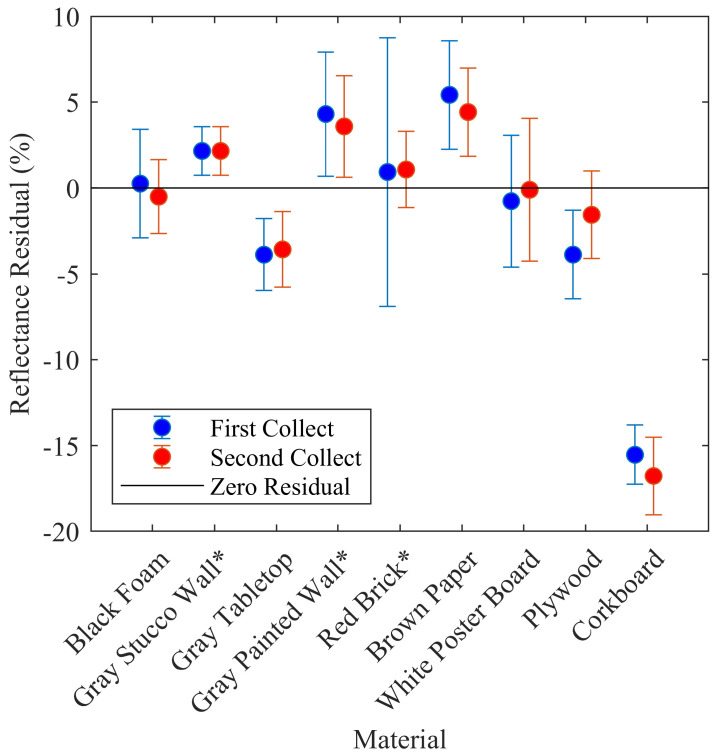
Temporal variation by material with 1σ error bars. The predicted reflectance residuals (vertical axis) are with respect to the reflectance measured with a spectroradiometer. Data from Sensor 1 shown.

For a calibration model to produce consistent results over time, the sensor measurements that are input into the model must also be temporally consistent. Therefore, the test targets were observed twice, separated by two months in time, and the mean predicted reflectance for each target was computed from all setups (spanning all target ranges and ambient light level conditions) and compared between collection dates. Sensor 1 results are illustrated in Figure 6, where the mean residual difference from the spectroradiometer measurement is indicated for each target on both collection dates along with 1σ error bars. Visual inspection of the error bars indicate agreement within ±1σ. Note that the three specular targets were excluded from this analysis.

On a more granular level, paired *t*-tests applied to multi-temporal matching setups for each target (same distance and nominal ambient light level but different collection date) indicate that only the Plywood and Corkboard targets for Sensor 1 are statistically different at 2σ. Similar results were obtained for Sensors 2–4, and are summarized in Table 6.

### 4.2. Cross-Sensor Performance

#### 4.2.1. Sensor Measurement Consistency

Comparison of the measurements reported by the four Evo 60 m sensors provides insight into the consistency of the manufacturing and factory calibration processes, which, in turn, informs the suitability of using a single trained radiometric calibration model for all four sensors. Distance, ambient light, and amplitude measurements having the same integration time were compared between the four sensors.

Beginning with distance, paired *t*-tests of the mean distances computed from individual setups collected in controlled ambient light conditions (indoors) indicate that only one sensor combination produces distance measurements not statistically different at 2σ—the Sensor 1 and Sensor 4 combination. For overall mean differences in distance computed from all setups, there is no difference (0.0 mm) between the distances reported by Sensor 1 and Sensor 4. The overall mean differences in distance for the remaining sensor combinations ranged from −17 to 17 mm (see Table 7).

Paired *t*-tests on the same group of indoor setups find that the ambient light measurements for all sensor combinations are statistically different at 2σ. The same is true for the amplitude measurements. See Table 7 for overall comparisons of these measurements generated from the mean of all setups for each sensor combination. Hence, we find that the sensor measurements are statistically different in almost all cases. The influence of these differences on the reflectance values produced by the sensor-specific radiometric calibration models is examined in the following section.

#### 4.2.2. Calibration Model Performance Consistency

The sensor measurement differences reported in the prior section indicate that differences in predicted reflectance will be realized if a single calibration model is applied to all four sensors. However, if the differences are small, a single model may be an attractive option for efficiently supplying calibration models for many sensors. The RMSE in predicted reflectance for the test target data resulting from cross-application of the four sensor-specific calibration models is given in Table 8. For clarity, the specular targets and Corkboard were not included in this analysis. As expected, the best performance is achieved when applying the sensor-specific models to their own test target observations (diagonal elements of the first four columns in Table 8). Among the three sensors purchased at the same time (Sensors 2–4), cross-application of the calibration models increased the RMSE values by factors approaching two. Model cross-application with the first sensor, which was purchased a year before the Sensors 2–4, increases RMSEs by factors approaching five. These results indicate that although a single trained calibration model can be applied to sensors manufactured at or near the same time to produce rough reflectance values, training sensor-specific calibration models is preferable.

Finally, in addition to the cross-application of the individual sensor calibration models, the use of a single neural network structure—but still training a unique set of neural network weights for each sensor—was examined. The network consisted of two layers with eight and four nodes, respectively, and used the same optimization and activation functions as used in the sensor specific network structures (BR optimization, tangent sigmoid activation function). The RMSE values for this common network structure are increased by factors of 1.25–1.5 compared to the sensor specific network structures, suggesting that a single network structure could be used to simplify the radiometric calibration of many sensors.

## 5. Conclusions

Radiometric calibration of traditional long-range topographic lidar sensors is well established. However, emerging low-cost, short-range lidar sensors may employ ranging methods that do not produce amplitude or intensity measurements appropriate for use with existing radiometric calibration techniques. A radiometric calibration model based on a simple feedforward neural network was therefore proposed to empirically model a complex and nonlinear relationship between the distance, amplitude, integration time, and ambient light measurements reported by a Terabee Evo 60 m sensor and target reflectance. In particular, the Evo 60 m reports return signal amplitude measurements that are not smoothly proportional to the return signal power and thus not appropriate for existing physical and empirical radiometric calibration models.

The neural network calibration model was demonstrated to predict target reflectance values consistent with spectroradiometer measurements at ±6% RMSE with precisions of ±5% or less. This performance is consistent with that of traditional lidar sensors and radiometric calibration models and was achieved with four different Evo 60 m sensors. The measurements reported by the four sensors were found to be most similar to one another for those sensors purchased at the same time (three of the sensors were purchased a year removed from the other sensor), but were still statistically different than each other at 95% confidence. Accordingly, each sensor performs best when using a radiometric calibration model whose structure was developed and network weights trained using its own measurements. However, using a common model structure, but still training a unique set of network weights for each sensor, was found to degrade the predicted reflectance values by only one or two percent in reflectance on average.

A neural network-based radiometric calibration model is a generic approach that may be applicable to other low-cost lidar sensors that report non-traditional amplitude or intensity values. Future research could also include determining a minimum set of calibration observations required to build a suitable model for the Evo 60 m sensor, and the extension of the radiometric calibration model beyond 14 m in range.

## Figures and Tables

**Table 1:** Terabee TeraRanger Evo 60 m specifications.

**General Properties**	
Wavelength	950 nm
Size	29 × 29 × 22 mm
Weight	12 g
Field of view (FOV)	1.7 degrees
Approximate Cost	124 ( $140)
**Ranging Characteristics**	
Range Envelope	0.5–60 m
Collection Rate	Up to 240 Hz
Range Resolution	5 mm (<14 m), 20 mm (>14 m)
Accuracy	±40 mm (<14 m), ±1.5% (>14 m)

**Table 2:** Field of View Size.

**Range (m)**	1	5	10	15	20	60
**Field of View Diameter (cm)**	3	15	30	45	60	180

**Table 3:** Test target measured reflectance at 950 nm.

Material	Reflectance (%)
Black Foam	5
Gray Stucco Wall *	19
Gray Tabletop	36
Gray Painted Wall *	45
Red Brick *	46
Brown Paper	59
White Poster Board	84
Plywood	87
Silver Door *	54
Brown Door *	56
White Painted Wall	82
Corkboard	86

* Outdoor material.

**Table 4:** Network hidden layer node counts.

Sensor	# Nodes (Layer 1)	# Nodes (Layer 2)
Sensor 1	8	3
Sensor 2	14	1
Sensor 3	7	4
Sensor 4	7	4

**Table 5:** Neural network performances for each sensors trained model (Units = reflectance %).

		Black Foam	Gray Stucco Wall *	Gray Tabletop	Gray Painted Wall *	Red Brick *	Brown Paper	White Poster board	Plywood	Silver Door *	Brown Door *	White Painted Wall	Corkboard
	Spectroradiometer Reflectance	5	19	36	45	46	59	84	87	54	56	82	86
Sensor 1	Mean Reflectance	5	21	32	49	47	64	84	84	83	64	134	70
	Δ	0 ${}^{†}$	2	−4	4	1 ${}^{†}$	5	0 ${}^{†}$	−3	29	8	52	−16
	RMSE	3	3	4	5	5	6	4	4	33	12	53	16
	σ	2.7	1.8	2.2	3.2	5.2	2.9	4	2.8	15.5	9.4	8.8	2.1
Sensor 2	Mean Reflectance	4	22	31	49	46	62	82	84	82	72	138	69
	Δ	−1	3	−5	4	0 ${}^{†}$	3	−2	−3	28	16	56	−17
	RMSE	2	3	5	5	4	4	5	4	32	23	57	17
	σ	1	1.5	1.6	3	4.4	2.5	4.4	2.7	15.9	17	10.4	2.9
Sensor 3	Mean Reflectance	3	22	32	49	48	64	85	84	96	59	138	70
	Δ	−2	3	−4	4	2	5	1 ${}^{†}$	−3	42	3	56	−16
	RMSE	2	3	4	4	3	6	4	4	119	6	57	16
	σ	0.6	1.8	1.3	2.3	2.6	3.3	4.4	2.4	111	5	10.2	2.2
Sensor 4	Mean Reflectance	4	22	32	49	47	63	83	85	89	70	132	70
	Δ	−1	3	−4	4	1	4	−1	−2	35	14	50	−16
	RMSE	2	4	5	5	4	5	5	4	39	24	51	16
	σ	1.7	2.3	2.1	3.8	3.3	3	4.7	3.3	18.3	19	9	3.5

* Material Collected Outdoor. ${}^{†}$ No Statistical Difference at 2σ.

**Table 6:** Mean difference in target reflectance (%) between temporally spaced data collections (June–August).

	Black Foam	Gray Stucco Wall *	Gray Tabletop	Gray Painted Wall *	Red Brick *	Brown Paper	White Poster board	Plywood	Corkboard
Sensor 1	0.8	0.4	−0.3	0.7	−0.1	1.0	0.7	−2.3 ${}^{†}$	1.2 ${}^{†}$
Sensor 2	0.7	−0.5	−0.7	0.6	−0.6	0.1	0.4	−1.6	1.4
Sensor 3	0.3	0.3	−0.6	0.4	0.0	0.5	−0.2	−1.4	0.6
Sensor 4	0.4	−1.0	−0.8	0.4	0.2	−0.2	0.4	−1.5	0.2

* Material collected outdoors. ${}^{†}$ Paired *t*-tests of multi-temporal setups indicate statistical difference at 2σ.

**Table 7:** Mean distance, ambient light, and amplitude differences for each sensor combination.

	S1–S2	S1–S3	S1–S4	S2–S3	S2–S4	S3–S4
Distance (mm)	−12.6	−16.6	0.0	−4.0	12.6	16.6
Ambient Light *	−10.1	16.1	−44.5	26.2	−34.5	−60.6
Amplitude ${}^{†}$	−45.3	−39.4	−31.2	5.9	14.0	8.1

* Unitless. Observed range was from 20 to 5793. ${}^{†}$ Unitless. Observed range was from 50 to 450.

**Table 8:** Cross-application of radiometric calibration models: Predicted reflectance RMSE (±%).

	Sensor 1 Model	Sensor 2 Model	Sensor 3 Model	Sensor 4 Model	Common Model Structure
Sensor 1	4	13	10	9	5
Sensor 2	18	4	6	7	5
Sensor 3	15	5	4	5	6
Sensor 4	12	6	4	4	5
